# Bronchial Dieulafoy: Diagnostic Utility of Endobronchial Ultrasound

**DOI:** 10.1002/rcr2.70282

**Published:** 2025-07-16

**Authors:** Chee Kiang Phua, Chee Kiang Tay, Carrie Kah Lai Leong

**Affiliations:** ^1^ Department of Respiratory and Critical Care Medicine Tan Tock Seng Hospital Singapore Singapore; ^2^ Department of Respiratory and Critical Care Medicine Singapore General Hospital Singapore Singapore

**Keywords:** bronchial Dieulafoy, endobronchial ultrasound, haemoptysis

## Abstract

We report a case of bronchial Dieulafoy presenting with haemoptysis, initially resembling an endobronchial tumour on bronchoscopy. This case highlights the critical role of EBUS in the assessment of suspicious endobronchial lesions and in preventing potentially life‐threatening complications from inadvertent biopsy.

## Graphical abstract

Bronchial Dieulafoy can mimic an endobronchial tumour and pose a risk of life‐threatening bleeding if biopsied. Endobronchial ultrasound (EBUS) confirmed the vascular nature of the lesion, guiding safe and effective management.

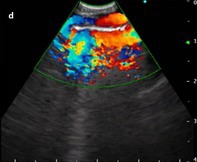

A 63‐year‐old male, chronic smoker, presented with intermittent episodes of haemoptysis for several days. Six months prior, he had similar episodes evaluated with chest computed tomography (CT) and nasoendoscopy, which were unremarkable. Flexible bronchoscopy performed 2 days after the haemoptysis episodes revealed a non‐pulsatile tubular endobronchial lesion at the right lower lobe anterior segment with no visible stigmata of recent bleeding (Figure 1a). Differentials included an endobronchial tumour versus bronchial Dieulafoy. A CT thoracic angiogram was not able to detect any abnormalities (Figure 1b). Due to concerns of airway bleeding, endobronchial ultrasound (EBUS) examination was performed via rigid bronchoscopy with view of sampling. Convex EBUS revealed a linear anechoic lesion (Figure 1c; Video 1) with arterial pulsation on colour doppler (Figure 1d). Radial EBUS also confirmed an anechoic lesion (Video 2). Based on these findings, a diagnosis of bronchial Dieulafoy was made and he successfully underwent bronchial angioembolization with resolution of haemoptysis (Figure 1e). Bronchial Dieulafoy is a rare cause of haemoptysis, and it can be easily misdiagnosed as an endobronchial tumour during bronchoscopy [1]. Convex EBUS uses a specialised bronchoscope equipped with an ultrasound transducer, whilst radial EBUS employs a flexible catheter with a rotating transducer, both allowing immediate confirmation of the diagnosis and averting a biopsy which can lead to catastrophic airway bleeding [1, 2].

**FIGURE 1:**
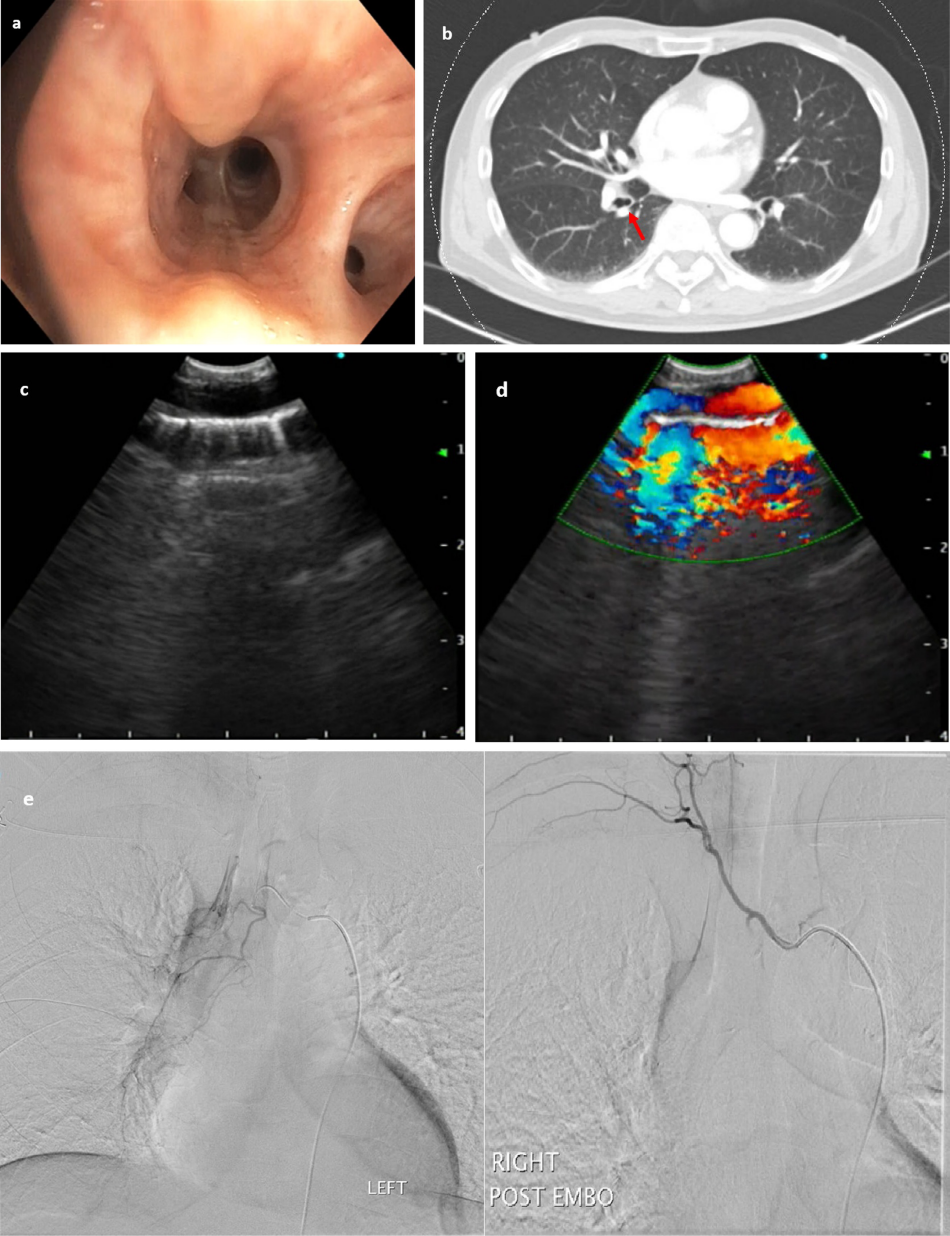
(a) Non‐pulsatile tubular‐like endobronchial lesion at the right lower lobe anterior segment. (b) CT thoracic angiogram with corresponding site of endobronchial lesion seen on bronchoscopy (red arrow) (c) Convex endobronchial ultrasound findings of a linear anechoic structure resembling a vessel. (d) Convex endobronchial ultrasound in colour doppler mode demonstrating blood flow within the linear anechoic structure. (e) Pre‐ and post‐bronchial embolisation images showing successful embolisation of right lower lobe bronchial arteries.

**VIDEO 1:** Convex EBUS showing blood flow within the endobronchial lesion on colour doppler. Video content can be viewed at https://onlinelibrary.wiley.com/doi/10.1002/rcr2.70282. Video content can be viewed at https://onlinelibrary.wiley.com/doi/10.1002/rcr2.70282.

**VIDEO 2:** Radial EBUS demonstrating an anechoic lesion adjacent to the ultrasound probe. Video content can be viewed at https://onlinelibrary.wiley.com/doi/10.1002/rcr2.70282. Video content can be viewed at https://onlinelibrary.wiley.com/doi/10.1002/rcr2.70282.

## Author Contributions

C.K.P. initiated the idea for manuscript submission and prepared the final draft. C.K.T. and C.K.L.L. acquired the clinical data. All authors have read and approved the final manuscript.

## Consent

The authors declare that written informed consent was obtained for the publication of this manuscript and accompanying images and attest that the form used to obtain consent from the patient complies with the Journal requirements as outlined in the author guidelines.

## Conflicts of Interest

The authors declare no conflicts of interest.

## Data Availability

Data sharing is not applicable to this article as no new data were created or analyzed in this study.
